# HEALTH SYSTEM PARTNERSHIPS IN INTERVENTION AND OBSERVATIONAL STUDIES: REFLECTIONS FROM AN EARLY-CAREER SCHOLAR

**DOI:** 10.1093/geroni/igad104.0423

**Published:** 2023-12-21

**Authors:** Zach Gassoumis

**Affiliations:** University of Southern California, Los Angeles, California, United States

## Abstract

The early knowledge base and interventions in the field of elder mistreatment were built by clinician researchers, leading to a natural research-to-practice pipeline. But with more non-clinical researchers steadily entering the field, care must be taken to forge strong research-practitioner partnerships, to three ends: 1) developing clinically informed research and intervention approaches, 2) recruiting clinically relevant samples, and 3) implementing findings into clinical practice. This process can be particularly challenging for early-career non-clinical researchers, who may lack strong partnerships on which to build their research. This presentation extracts lessons for early career scholars by providing overviews of the development of and findings from two studies. The Comprehensive Older Adult Caregiving Supports (COACH) EM prevention program was delivered in partnership with a managed care health system, with referrals received from health system providers. The Better Together study of dementia care groups (i.e., persons living with dementia and their care partners) received referrals from clinicians in mixed managed care/fee-for-service settings, county health facilities, and community partners. Drawing from the principles of community-engaged research, the presentation highlights the relevance of various engagement approaches to elder mistreatment research, including: 1) partnering with senior researchers with existing networks; 2) taking the time to learn the health system’s and clinicians’ operational systems, pressures, and constraints; 3) honoring practitioner expertise in building research projects and developing research questions; 4) prioritizing meaningful reciprocal engagement, through offering topical or research presentations, funding, co-authorship, etc.; and 5) educating health system administrators about the nuances of conducting elder mistreatment research.

